# A Unique Compensatory Mechanism for Total Pulmonary Vein Occlusion Post Atrial Fibrillation Catheter Ablation Visualized by Multimodality Imaging

**DOI:** 10.1155/2020/9673958

**Published:** 2020-09-23

**Authors:** Ayman R. Fath, Amro Aglan, Luis R. Scott, Clinton E. Jokerst, Hemalatha Narayanasamy, Farouk Mookadam, Nawfal Mihyawi, Nithin R. Venepally, Sudheer Konduru, Reza Arsanjani

**Affiliations:** ^1^Department of Cardiovascular Diseases, Mayo Clinic Arizona, USA; ^2^Beth Israel Deaconess Medical Center, Harvard Medical School, Boston, MA, USA; ^3^Department of Radiology, Mayo Clinic Arizona, USA; ^4^Department of Internal Medicine, Creighton University Arizona Health Alliance, USA

## Abstract

Pulmonary vein (PV) stenosis is a rare and serious complication of radiofrequency catheter ablation (RFCA) for atrial fibrillation. However, it can be asymptomatic or mildly symptomatic depending on the severity of the stenosis and the development of compensatory mechanisms. This study provides a detailed description and visualization of a unique type of venous collaterals that bypass the PV stenosis and drain directly in the left atrium alleviating PV stenosis sequelae. This study reports a case of a 61-year-old male who presented with mild dyspnea and fatigue 3 years post atrial fibrillation RFCA. After a thorough evaluation of the case, a redo-ablation was planned. As a part of the preablation workup, a transesophageal echocardiography (TEE), a ventilation-perfusion (V/Q) scan of the lungs, and a chest computed tomography angiogram (CTA) were performed. The TEE revealed total obstruction of the left superior PV, with no color Doppler flow detected. It also showed evidence of multiple collateral flows at the os of the left superior PV. The V/Q scan showed a large perfusion defect involving the entire left upper lobe consistent with a compromised left upper PV flow. The CTA with 3D volume rendering revealed the total occlusion of the left superior PV at its ostium. Moreover, the scan confirmed the pulmonary venous drainage via small collateral channels that was suggested by the TEE.

## 1. Introduction

Atrial fibrillation (AF) is the commonest atrial arrhythmia resulting from one or multiple wavelets propagating in different directions [1]. Pulmonary veins (PVs) and PV ostia are the primary foci of these wavelets that trigger AF [1]. Radiofrequency catheter ablation (RFCA) with pulmonary vein isolation is recommended for eligible patients with symptomatic atrial fibrillation [1, 2]. Severe PV stenosis is a rare complication of RFCA with PV isolation (~1%), and the incidence of symptomatic severe PV stenosis requiring treatment is even less common [3]. Symptomatic total PV occlusion may develop over years and has been reported in the literature. We present a case of total PV occlusion following RFCA that remained mildly symptomatic due to partial compensation from collateral circulation formation which bypassed the occluded segment of PV. The collateral circulation flows were identified with transesophageal echocardiography (TEE) and confirmed with chest computed tomography angiogram (CTA).

## 2. Case Report

A 61-year-old gentleman presented with mild dyspnea and fatigue. He was noted to be in AF on pacemaker interrogation and ECG. His history was significant for paroxysmal AF diagnosed three years prior when he was admitted to the hospital with embolic stroke. The AF was initially managed with a rate-lowering calcium channel blocker and flecainide coupled with a direct oral anticoagulant. Despite optimized medical therapy, he continued to be symptomatic; therefore, he underwent antral RFCA two months later. The ablation consisted of PV isolation using radiofrequency catheter ablation. Complete isolation with entrance and exit block of the left side PV was achieved. Due to the rise in esophageal temperature during radiofrequency application, the right-sided veins were not completely isolated. Complex fractionated atrial electrogram ablations were targeted for ablation in both the left and right atria, as well as the ostium of the coronary sinus. The patient had postablation prolonged sinus pause that required dual-chamber pacemaker implantation. At presentation, his device interrogation revealed an AF burden of 60% with several periods of rapid ventricular response. In light of the ongoing symptoms, a redo-ablation was planned. The patient has no pertinent past medical, social, or family history. Physical examination showed irregular heart rhythm but was otherwise unremarkable.

**Figure 1:**
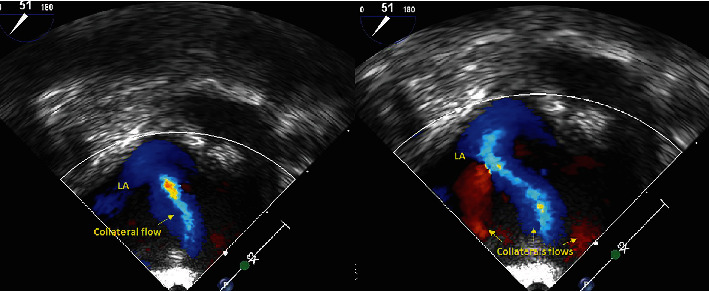
TEE shows multiple collateral flows at the os of the left superior PV with peak velocity of the predominant flow of 45 cm/s.

As a part of the preablation workup, a TEE, a ventilation-perfusion (V/Q) scan, and a chest CTA were performed. The TEE revealed total obstruction of the left superior PV, with no color Doppler flow detected. It also interestingly showed evidence of multiple collateral flows at the os of the left superior PV with a peak velocity of the predominant flow of 45 cm/s (Figure 1).

**Figure 2:**
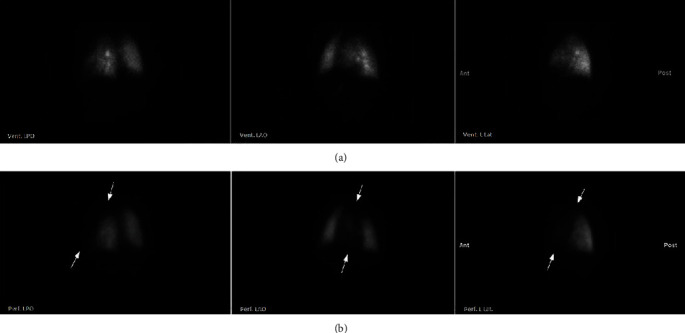
Lung V/Q scan showing absent perfusion of the left upper pulmonary lobe and lingula (arrows) based on the comparison of the relatively (a) normal ventilation to the (b) abnormal perfusion in the left posterior oblique (LPO), left anterior oblique (LAO), and left lateral (L lat.) projections.

The V/Q scan of the lungs showed a large mismatched perfusion defect involving the entire left upper lobe and lingula and relatively decreased but preserved ventilation in the left upper lung, consistent with a compromised left upper PV flow (Figure 2).

**Figure 3:**
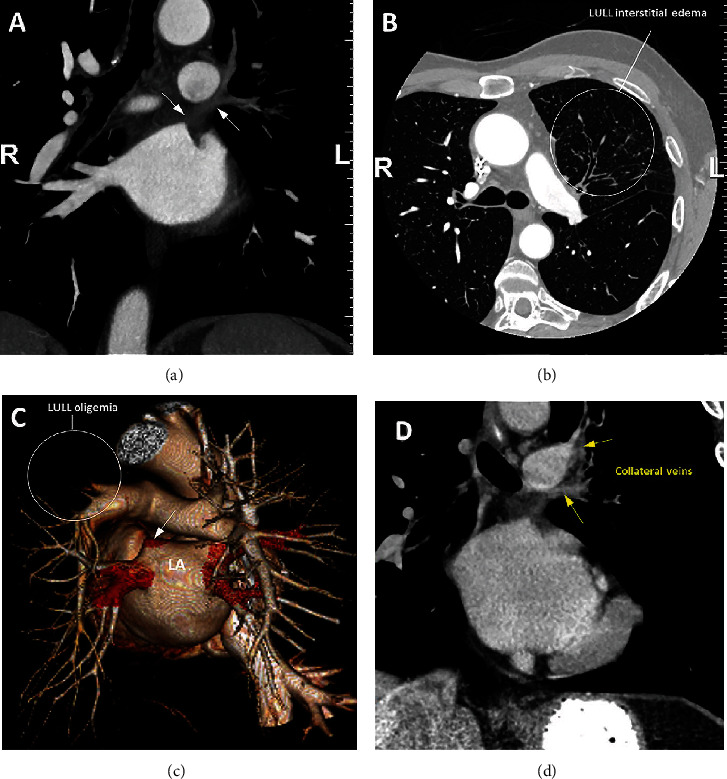
Coronal oblique view (a), axial view (b), and volume-rendered 3D reconstruction (c) from an arterial phase CT angiogram demonstrate absence or stump of the left superior pulmonary vein (white arrows) and left upper lobe oligemia ((c), circled) and chronic interstitial edema ((b), circled). Coronal oblique vascular reconstruction from a delayed venous phase CT angiogram (d) demonstrates small collateral draining veins (yellow arrows). LULL = left upper lung lobe.

The CTA of the chest with 3D volume rendering showed the total occlusion of the left superior PV at its ostium and confirmed residual pulmonary venous drainage via small collateral channels. It also showed chronic interstitial edema, fibrosis, and oligemia within the left upper lobe. The remainder of the lungs appeared clear (Figure 3).

## 3. Discussion

Treatment of AF includes pharmacologic therapy with rate control, rhythm control, and/or invasive therapy with catheter ablation. Cryoablation and RFCA are approved treatment modalities for patients with symptomatic paroxysmal or persistent AF refractory or intolerant to medical treatment [2, 4]. Most of the ectopic beats in AF arise from PVs and PV ostia; therefore, PV isolation is the main focus of ablation procedures [1, 4]. Pulmonary vein stenosis is the most frequently reported long-term complication post-RFCA. The mechanism of such is likely due to endocardial injury causing scar and cicatrization with intimal hyperplasia and fibrosis associated with the healing process [5]. However, its incidence has been getting lower over time due to increased awareness and improvement in ablation techniques [3].

Symptoms of PV stenosis depend on the ipsilateral lung perfusion reflecting the ability to develop compensatory mechanisms [6]. These compensatory mechanisms include alteration of pulmonary hemodynamics and redistribution of blood flow with neovascularization of the ipsilateral lung. This makes the venous drainage of the affected area mostly dependent on the ipsilateral veins draining the healthy lobes [6]. Di Biase et al. [6] completed a physiologic evaluation with V/Q scanning of 18 patients with PV occlusion among 1780 patients who underwent AF ablation from 1999 to 2004. The study showed that all patients with severe symptoms had relative perfusion < 25% of the ipsilateral lung, whereas all asymptomatic patients and those with mild symptoms were above this value. Our case supports this study findings; the patient was mildly symptomatic with relative perfusion of 50% of the ipsilateral lung, which was likely due to compensatory changes as described above. Furthermore, our patient has developed additional compensatory collateral veins bypassing the occluded PV and draining directly in the left atrium. These collateral veins are believed to contribute to more symptom relief.

Yun et al. [7] discussed the spontaneous improvement in most cases of PV stenosis due to compensatory hemodynamics. They reported hemodynamic changes detected by time-resolved contrast-enhanced magnetic resonance angiography (TR-MRA) in a patient with right PV stenosis following RFCA of AF. TR-MRA demonstrated prominent systemic arteries in the right thorax connected with the right pulmonary artery, suggesting pulmonary to systemic arterial collaterals. In addition, on a velocity-encoded cine image, the flow direction of the right pulmonary artery was reversed in the diastolic phase resulting in drainage of oxygen-rich blood back to the contralateral pulmonary artery. These hemodynamic changes were similar to those seen in congenital unilateral PV atresia [8]. Our study shows a different type of collateral flow that developed on the venous side, detected by TEE and confirmed with CTA.

In conclusion, PV stenosis is a serious complication of RFCA. Its incidence has been reported to range from 3% to 42% depending on the technique used and assessment method [9]. It is associated with significant morbidity and complications that may require revascularization or even partial lobectomy. Diagnosis of PVS can be challenging due to its nonspecific symptoms; therefore, it requires a low index of suspicion. The spectrum of symptoms can vary from persistent cough to severe dyspnea and significant hemoptysis. Nonetheless, some patients may remain asymptomatic or mildly symptomatic due to the development of compensatory mechanisms. Different compensatory mechanisms have been well documented in the literature including alterations of pulmonary hemodynamics and redistribution of blood flow. In our case report, we demonstrate another compensatory drainage mechanism via collateral veins connected directly to the left atrium. We also illustrate the utility of a detailed TEE and CTA with 3D volume rendering in identifying these collateral veins.

## Supplementary Materials

Supplementary Materials The following supplementary material is available online. Movie Clips: Video 1: TEE shows total obstruction of the left superior pulmonary vein (LSPV), with no color Doppler flow detected. It also shows collateral flow at the os of the LSVP (yellow arrow).
